# Hospital volume and outcome in inguinal hernia repair: analysis of routine data of 133,449 patients

**DOI:** 10.1007/s10029-019-02091-8

**Published:** 2019-11-30

**Authors:** M. Maneck, F. Köckerling, C. Fahlenbrach, C. D. Heidecke, G. Heller, H. J. Meyer, U. Rolle, E. Schuler, B. Waibel, E. Jeschke, C. Günster

**Affiliations:** 1 AOK Research Institute (WIdO), Berlin, Germany; 2 Department of Surgery and Center for Minimally Invasive Surgery, Academic Teaching Hospital of Charité Medical School, Vivantes Hospital, Berlin, Germany; 3 Federal Association of AOK, Berlin, Germany; 4 Department of General, Visceral, Thoracic and Vascular Surgery, University of Greifswald, Greifswald, Germany; 5 Department of Medicine, University of Marburg, Marburg, Germany; 6 German Society of Surgery, Berlin, Germany; 7 Department of Pediatric Surgery and Pediatric Urology, University of Frankfurt/Main, Frankfurt/Main, Germany; 8 Department of Quality Management, Helios Hospitals, Berlin, Germany; 9 Medical Review Board of the Social Health Insurance Funds Baden-Württemberg, Freiburg, Germany

**Keywords:** Inguinal hernia, Recurrence, Volume-outcome, Case load, Routine data, Postoperative complications

## Abstract

**Introduction:**

Inguinal hernias are repaired using either open or minimally invasive surgical techniques. For both types of surgery it has been demonstrated that a higher annual surgeon volume is associated with a lower risk of recurrence. This present study investigated the volume-outcome implications for recurrence operations, surgical complications, rate of chronic pain requiring treatment, and 30-day mortality based on the hospital volume.

**Materials and methods:**

The data basis used was the routine data collected throughout the Federal Republic of Germany for persons insured by the Local General Sickness Fund “AOK” who had undergone inpatient inguinal hernia repair between 2013 and 2015. Complications were recorded by means of indicators. Hospitals were divided into five groups on the basis of the annual caseload volume: 1–50, 51–75, 76–100, 101–125, and ≥ 126 inguinal hernia repairs per year. The effect of the hospital volume on the indicators was assessed using multiple logistic regression.

**Results:**

133,449 inguinal hernia repairs were included. The incidence for recurrence operations was 0.95%, for surgical complications 4.22%, for chronic pain requiring treatment 2.87%, and for the 30-day mortality 0.28%. Low volume hospitals (1–50 and 51–75 inguinal hernia repairs per year) showed a significantly increased recurrence risk compared to high volume hospitals with ≥ 126 inguinal hernia repairs per year (odds ratio: 1.53 and 1.24). No significant correlations were found for the other results.

**Conclusions:**

The study gives a detailed picture of hospital care for inguinal hernia repair in Germany. Furthermore, it was noted that the risk of hernia recurrence decreases in line with a rising caseload of the treating hospital.

## Introduction

Worldwide, more than 20 million patients undergo groin hernia repair per year [1]. In Germany alone, 170,000 inpatient inguinal hernia repairs were carried out in 2016 [2]. Hence, inguinal hernia repair ranks among the 20 most common surgical procedures performed in German hospitals [2]. A systematic review of the perioperative complications associated with inguinal hernia repair based on 39 studies with 571,445 patients identified a rate of 2.9% (*n* = 16.482/577.445) [3]. The most common complications were bleeding (0.86%), surgical site infections (0.48%), and other complications (0.41%) [3]. The chronic pain rate given in the international guidelines is 0.5–6.0% [1]. The surgical technique, gender, and size of the hernia defect have a decisive impact on the rate of chronic pain requiring treatment [4–6].

Of the total collective of repaired inguinal hernias, the proportion of recurrent inguinal hernias is 11% [7].

In addition to the hernia- and patient-related influencing factors on the outcome of inguinal hernia surgery, those related to the surgeon volume and hospital volume are also being increasingly investigated [8–14]. For example, one analysis of data from the Danish Hernia Database revealed that hospitals with less than 50 inguinal hernia repairs per year were found to have a significantly higher rate of recurrence operations (9.97% vs 6.06%; *p* < 0.0001) compared with hospitals with 50 and more operations [9]. Another analysis by the Statewide Planning and Research Cooperative System in the USA did not find any difference in outcome for hospitals with less than 140 inguinal hernia repairs compared with hospitals with 140 and more operations [14].

All the other aforementioned studies [8, 10–13] focused on the specific volume of an individual surgeon and its corresponding impact on the outcome. These studies have demonstrated that, as regards the surgeon volume, surgeons with higher caseloads have a lower recurrence rate [8, 10–13].

That thus raises the issue of whether for those hospitals with several surgeons and various surgical techniques differences can also be identified at a hospital level in the outcomes in relation to the caseload. That issue is particularly interesting from a patient’s perspective since the choice of hospital is still often not based on the surgeon or surgical technique. A direct link between a hospital’s number of inguinal hernia repairs and the outcome would serve as a rough guide to choosing a hospital, as in Germany, data on hospital caseload is publicly available.

The present study of routine administrative data from the German Local General Sickness Fund “AOK” [15] aimed to identify whether a correlation could be identified between the hospital volume and outcome.

## Materials and methods

### Data basis

The analyses were based on anonymized routine data of the German Local General Sickness Fund (AOK). These included diagnoses and procedures related to hospital care, drug prescriptions as well as insured persons’ master data such as age, gender and survival status.

**Fig. 1:**
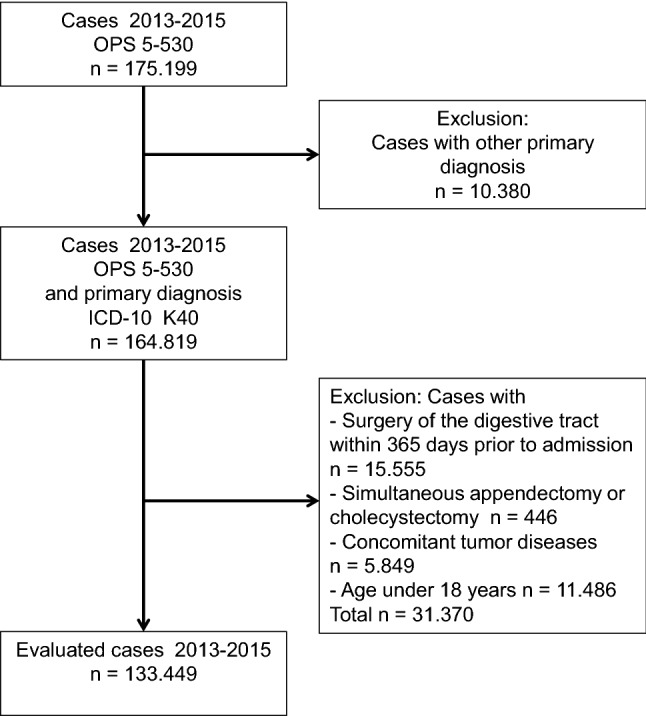
Applied criteria and stepwise case exclusion

Included in the analysis were hospital inpatients and outpatients who during the initial hospital stay underwent inguinal hernia repair (Operations and Procedures Key [OPS]: 5-530) between 2013 and 2015 and for whom inguinal hernia (International Statistical Classification of Diseases and Related Health Problems [ICD]-10: K40) was documented as primary diagnosis. Patients under 18 years of age, with simultaneous appendectomy (OPS: 5-470, 5-471), cholecystectomy (OPS: 5-511) or cancer disease (ICD-10: C00–C97, D00–D09, D37–D48; OPS: 8-54) as well as patients who had undergone surgery of the digestive tract (OPS: 5-42 to 5-54) within 365 days prior to hospital admission were excluded (Fig. 1). The applicable criteria had been formulated for the inguinal hernia repair service area by the Abdominal Surgery Expert Panel within the framework of the quality assurance with routine data (QSR) project of the Scientific Institute of the AOK (WIdO) [16].

For each patient a follow-up period of 365 days from discharge after the initial hospital stay was reviewed. Additional inguinal hernia operations within this period were only assessed as outcomes. In compliance with data protection regulations, the AOK data used were anonymized such that the patient’s identity was not known and could not be determined.

### Endpoints

The endpoints employed corresponded to the definitions of the quality indicators used for inguinal hernia repair, which had been compiled by the Scientific Institute of the AOK [16]. The endpoints analyzed were 30-day mortality, recurrence operations within 91–365 days, chronic pain requiring treatment within 365 days, and surgical complications within 90 days.

Recurrence operations within 91–365 days were documented on the basis of inguinal hernia repairs (OPS: 5-530) performed on the same side of the body as the primary repair procedure. Reoperations within 90 days were not documented for that indicator since they tended to relate to technical defects or complications. Such events were ascribed to the endpoint “surgical complications within 90 days”.

Chronic pain requiring treatment within 365 days was documented on the basis of continuous drug prescriptions. A new case of continuous pain management was assumed if patients had been prescribed at least 20 defined daily doses [DDD] of analgesics in at least three-out-of-four quarters (Anatomic Therapeutic Chemical classification [ATC]: M01, N02A N02B) and had not been on continuous pain management prior to surgery (≥ 20 DDDs in ≥ 3-out-of-4 quarters before admission).

Surgical complications within 90 days consisted of reoperations in the abdominal and inguinal region, urologic reoperations, wound infections, bleeding requiring reoperation as well as resuscitation, pulmonary embolism and thrombosis, and general complications in association with surgical procedures [16]. The applicable ICD-10 and OPS codes are illustrated in Tables 1 and 2.

**Table 1:** Inclusion diagnoses for endpoint surgical complications, secondary diagnoses on initial hospital admission (IA) and primary diagnoses on readmission within the specified period of time (RA)

ICD-10	Description	Time period
N45	Orchitis and epididymitis	IA + RA 90 days
N49.2	Inflammatory disorders of scrotum	RA 90 days
N50.0	Atrophy of testis	RA 90 days
N50.1	Vascular disorders of male genital organs	RA 90 days
T81.0^a^	Hemorrhage and hematoma as a complication of a procedure, not elsewhere classified	IA + RA 7 days
A41	Sepsis, unspecified	RA 14 days
K65	Peritonitis	RA 14 days
T81.4	Infection following a procedure, not elsewhere classified	IA + RA 14 days
I26	Pulmonary embolism	IA + RA 30 days
I80.1	Thrombosis, phlebitis and thrombophlebitis of femoral vein	IA + RA 90 days
I80.2	Thrombosis, phlebitis and thrombophlebitis of other deep vessels of lower extremities	IA + RA 90 days
I80.3	Thrombosis, phlebitis and thrombophlebitis of other extremities, unspecified	IA + RA 90 days
I82.2	Embolism and thrombosis of vena cava	IA + RA 90 days
K56	Paralytic ileus and intestinal obstruction, no hernia	RA 90 days
K63.1	Perforation of intestine (nontraumatic)	IA + RA 90 days
K66.0	Peritoneal adhesions	RA 90 days
K66.1	Hemoperitoneum	RA 90 days
K91.3	Postprocedural intestinal obstruction	IA + RA 90 days
K91.83	Anastomosis and suture insufficiency following surgery elsewhere in the digestive tract	IA + RA 90 days
K91.88	Other disorders of the digestive system following medical measures, not elsewhere classified	IA + RA 90 days
K91.9	Disorder of the digestive system following medical measures, unspecified	IA + RA 90 days
T81.1	Shock during or resulting from a procedure, not elsewhere classified	IA + RA 90 days
T81.2	Accidental puncture or laceration during a procedure, not elsewhere classified	IA + RA 90 days
T81.3	Disruption of surgical wound, not elsewhere classified	IA + RA 90 days
T81.5	Foreign body accidentally left in body cavity or operation wound following a procedure	IA + RA 90 days
T81.6	Acute reaction to foreign substance accidentally left during a procedure	IA + RA 90 days
T81.7	Vascular complications following a procedure, not elsewhere classified	IA + RA 90 days
T81.8	Other complications of procedures, not elsewhere classified	IA + RA 90 days
T81.9	Unspecified complication of procedure	IA + RA 90 days

^a^Only if simultaneous presence of OPS 5-541.[0, 1, 2, 3], 5-549.5, 5-892.1[b, c], 5-916.a[0, 3], 5-983, 8-159.x, 8-190

5-892.0[b,c] or 5-896 [1, 2][b, c] within 1–7 days following surgery

**Table 2:** Inclusion procedures for endpoint surgical complications within the specified time period following surgery (initial hospital stay and readmissions) or only after readmission (RA) within specified time period

OPS	Description	Time period
(a)		
5-388.3	Suture of blood vessels: Aorta	0–90 days
5-388.5	Suture of blood vessels: Abdominal and pelvic	0–90 days
5-388.6	Suture of blood vessels: Visceral arteries	0–90 days
5-388.7	Suture of blood vessels: Femoral arteries	0–90 days
5-388.9	Suture of blood vessels: Deep veins	0–90 days
5-388.a	Suture of blood vessels: Superficial veins	0–90 days
5-467.0	Other reconstruction of intestines: Suture (following injury)	0–90 days
5-467.1	Other reconstruction of intestines: Repair of intestinal fistula, open surgery	1–90 days
5-467.5	Other reconstruction of intestines: Revision following anastomosis	RA 90 days
5-469.0	Other operation of intestines: Decompression	RA 90 days
5-469.1	Other operation of intestines: Adhesiolysis	RA 90 days
5-469.2	Other operation of intestines: Adhesiolysis	RA 90 days
5-469.e	Other operation of intestines: Injection	RA 90 days
5-530^a^	Inguinal hernia repair	1–90 days
5-540.0	Incision of abdominal wall: Exploration	RA 90 days
5-540.1	Incision of abdominal wall: Extraperitoneal drainage	RA 90 days
5-540.2	Incision of abdominal wall: Removal of a foreign body	RA 90 days
5-541.0	Laparotomy and opening of retroperitoneum: Explorative laparotomy	1–90 days
5-541.1	Laparotomy and opening of retroperitoneum: Laparotomy with drainage	1–90 days
5-541.2	Laparotomy and opening of retroperitoneum: Relaparotomy	RA 90 days
5-541.3	Laparotomy and opening of retroperitoneum: Second-look laparotomy (programmed relaparotomy)	RA 90 days
5-541.4	Laparotomy and opening of retroperitoneum: Placement of temporary abdominal wall closure	RA 90 days
5-545.0	Closure of abdominal wall and peritoneum: Secondary closure of abdominal wall (for postoperative wound dehiscence)	1–90 days
5-549.0	Other abdominal operations: Removal of a foreign body from abdominal cavity	1–90 days
5-549.5	Other abdominal operations: Laparoscopy with drainage	1–90 days
5-590.2	Incision and excision of retroperitoneal tissue: Drainage, retroperitoneal	RA 90 days
5-590.3	Incision and excision of retroperitoneal tissue: Drainage, pelvic	RA 90 days
5-892.1[b,c]	Other incision of the skin and subcutaneous tissues: Drainage ((abdominal/inguinal/genital regions)	1–90 days
5-892.3[b,c]	Other incision of the skin and subcutaneous tissues: Implantation of a drug delivery system (abdominal/inguinal/genital regions)	1–90 days
5-895.0[b,c]	Radical and extensive excision of diseased tissue from the skin and subcutaneous tissues: Without primary wound closure ((abdominal/inguinal/genital regions)	1–90 days
5-895.1[b,c]	Radical and extensive excision of diseased tissue from the skin and subcutaneous tissues: Without primary wound closure, under histographic control (abdominal/inguinal/genital regions)	1–90 days
5-895.2[b,c]	Radical and extensive excision of diseased tissue from the skin and subcutaneous tissues: With primary wound closure	1–90 days
5-895.3[b,c]	Radical and extensive excision of diseased tissue from the skin and subcutaneous tissues: With primary wound closure, under histographic control (abdominal/inguinal/genital regions)	1–90 days
5-916.a0^b^	Temporary soft tissue coverage: Placement or replacement of a system for vacuum sealing of the skin and subcutaneous tissues	2–90 days
5-916.a3^b^	Temporary soft tissue coverage: Placement or replacement of a system for vacuum sealing of open abdomen	2–90 days
5-983	Reoperation	1–90 days
8-153	Therapeutic percutaneous puncture of abdominal cavity	1–90 days
8-159.x	Other therapeutic percutaneous puncture: Unspecified	1–90 days
8-176	Therapeutic irrigation of the abdominal cavity with indwelling drain and temporary abdominal wall closure	1–90 days
8-190^b^	Special bandaging techniques	2–90 days
5-578.0	Other plastic reconstruction of urinary bladder: Suture (following injury)	0–30 days
5-622	Orchidectomy	1–90 days
5-639.1	Other operations of spermatic cord, epididymis and vas deferens: Incision of spermatic cord	RA 90 days
5-639.2	Other operations of spermatic cord, epididymis and vas deferens: Adhesiolysis of spermatic cord	RA 90 days
5-639.x	Other operations of spermatic cord, epididymis and vas deferens: Unspecified	RA 90 days
8-132.3	Manipulations of urinary bladder: Irrigation, continuous	RA 30 days
8-800	Transfusion of whole blood, erythrocyte concentrate and thrombocyte concentrate	0–7 days
5-896.1[b,c]	Surgical wound toilet (wound debridement) with removal of diseased tissue from the skin and subcutaneous tissues: Extensive ((abdominal/inguinal/genital regions)	1–14 days
5-896.2[b,c]	Surgical wound toilet (wound debridement) with removal of diseased tissue from the skin and subcutaneous tissues: Extensive with implantation of a drug delivery system (abdominal/inguinal/genital regions)	1–14 days
8-771	Cardiac or cardiopulmonary resuscitation	0–30 days
8-772	Operative resuscitation	0–30 days
8-779	Other resuscitation measures	0–30 days

^a^Procedure on the same side of the body as the primary procedure

^b^No simultaneous presence of decubitus ulcer (ICD-10 L89) or leg ulcer (ICD-10 I70.2 [3, 4], I83.[0,2], L97) at baseline

### Statistical analysis

Since only data belonging to AOK-insured persons were available, the hospital volume was projected based on the AOK inguinal hernia repair cases described above and on a hospital’s total proportion of AOK cases. Based on each hospital’s annual caseload, the AOK cases were divided into five categories (1–50, 51–75, 76–100, 101–125 and ≥ 126 cases) on an annual basis. In each case descriptive statistics were calculated as a total as well as for the individual volume categories. Trends in respect to the volume categories were verified with the Cuzick test using a significance level of 5%. All key figures given refer in each case to the evaluable caseload. Patients without complete follow-up and who did not experience a complication event within the follow-up period were censored.

The effect of the hospital volume on the endpoints was calculated using multiple logistic regression models. Hospitals with the highest caseloads were used as reference category. The regression models included the hospital volume while also making adjustment for age, gender, surgical technique, comorbidities, and other risk factors such as recurrence status, incarceration, extent of the operation, and preoperative medication. All parameters were defined as dichotomous variables. Age was defined on the basis of dichotomous categorical variables dividing the AOK cases into quintiles. Comorbidities were identified as per the Elixhauser definitions [17]. Cancer diseases were not considered since patients with such disease were not included in the data set. The risk factor obesity was categorized in accordance with the breakdown given in the ICD-10 catalog as grade I (BMI ≥ 30 and < 35), II (BMI ≥ 35 and < 40), and III (BMI ≥ 40) obesity as well as obesity grade unspecified. The surgical techniques were divided into three main groups: open mesh procedure (OPS: 5-530 [3, 7].[0, *x*]), minimally invasive mesh procedure (OPS: 5-530 [3, 7] [1, 2]), and meshfree procedure (OPS: 5-530.[0, 1, 2, 4, 5, 8, *x*, *y*]). Recurrence status, incarceration, extent of operation, and other risk factors were defined in accordance with the AOK’s Quality Assurance of Inpatient Care with Routine Data (QSR) specifications [16]. Model selection was performed using a stepwise backward algorithm based on a model with all adjustment variables. The calculated model was then expanded to include the factors influencing the hospital volume. All models were examined for collinearity by calculating variance inflation factors (VIF). The VIF of a covariate measures the extent of collinearity present [18–22]. If no collinearity is present for a covariate, its VIF equals 1. As the collinearity increases the VIF increases. In the literature different VIF thresholds are used. Kleinbaum et al. [18] and Montgomery et al. [19] used a value of 10 while Zuur et al. [22] used a more stringent value of 3.

For endpoints significantly impacted by the hospital volume the number needed to treat (NNT) was calculated with the logistic model [23]. If theoretically patients were reassigned from one category to the reference category, the NNT is the number of patients needed to be treated to prevent one complication. The total number of preventable complications of a category is equal to the quotient of the respective caseload and NNT.

All evaluations were performed with the software STATA14.2 (StataCorp, College Station, Texas).

## Results

The investigated data set comprised 133,449 AOK cases from 1060 hospitals for the years 2013–2015. These include all types of hospitals, e.g., government hospitals, private hospitals, university hospitals, and small community hospitals, in which AOK-insured patients were treated. The median patient age was 59 years (IQR: 47–73). The proportion of female patients was 11.6%. Detailed descriptive statistics are given in Table 3. Hernia recurrence present on admission was observed in 11.7% of patients and incarceration in 9.4%. The proportions of patients with incarceration, emergency, preoperative antithrombotic therapy as well as with comorbidities such as cardiac arrhythmia, COPD, renal failure or renal insufficiency significantly declined in line with rising hospital caseload volumes. By contrast, the proportion of patients with bilateral procedure or simultaneous umbilical hernias increased. In total, 55.9% of patients were treated with a minimally invasive mesh procedure, 37.9% with open mesh procedure, and 6.2% with open suture procedure. In line with increasing caseload volumes the proportion of minimally invasive mesh procedures rose from 40.1% in hospitals with the lowest caseload to 59.4% in those with the highest caseload. At the same time, the proportion of open mesh procedures dropped from 49.3 to 34.8% and the proportion of open suture procedures from 10.6 to 5.8%.

**Table 3:** Descriptive statistics of the included AOK cases (2013–2015), presented as a total figure and in accordance with volume categories (I: 1–50, II: 51–75, III: 76–100, IV: 101–125, V: ≥ 126 inguinal hernia repairs per year)

	Total	I	II	III	IV	V
Caseloads, age and gender						
Annual caseload	–	1–50	51–75	76–100	101–125	≥ 126
AOK cases (*N*)	133.449	4.586	12.105	17.985	20.776	77.997
Age (median; IQR)	59 (47–73)	60 (49–74)	61 (48–74)	60 (48–74)	60 (48–73)	59 (47–73)
Gender (female, %)	11.33	11.16	10.04	11.15	11.20	11.61
Risk factors and surgical technique (%)						
Recurrence present on admission	11.66	12.10	11.31	12.12	11.77	11.55
Incarceration	9.42	10.64	11.93	11.10	9.86	8.45
Gangrene	0.44	0.48	0.45	0.50	0.46	0.42
Emergency	4.08	4.62	5.15	4.96	4.36	3.60
Bilateral procedure	15.61	9.97	11.55	12.64	14.56	17.53
Intestinal procedure	0.35	0.44	0.35	0.42	0.32	0.34
Simultaneous repair of umbilical hernia	5.35	3.99	4.38	4.73	4.93	5.84
Simultaneous repair of femoral hernia	0.38	0.44	0.36	0.36	0.37	0.39
Simultaneous repair of incisional hernia	0.32	0.26	0.28	0.33	0.32	0.32
Outpatient repair	11.21	13.06	12.43	11.08	11.79	10.79
Open suture procedure	6.20	10.60	7.45	7.09	5.37	5.76
Open mesh procedure	37.92	49.30	43.89	41.03	40.85	34.82
Minimally invasive mesh procedure	55.89	40.10	48.66	51.88	53.78	59.42
Preoperative medication (%)						
Immunosuppressants	0.74	0.76	0.70	0.71	0.73	0.75
Systemic glucocorticoids	1.70	1.92	1.76	1.85	1.55	1.69
Antithrombotics	13.10	14.17	14.46	13.88	13.72	12.48
Treatment for chronic inflammatory bowel disease	0.32	0.31	0.37	0.36	0.34	0.31
BMI and Elixhauser comorbidities^a^ (%)						
Obesity grade, unspecified	0.30	0.44	0.23	0.37	0.26	0.30
Grade I obesity	2.79	3.47	2.73	2.66	2.81	2.79
Grade II obesity	0.93	1.02	0.98	0.86	1.04	0.91
Grade III obesity	0.36	0.57	0.40	0.36	0.34	0.35
Hypertension, no complications	31.34	34.89	34.69	33.37	32.36	29.87
Cardiac arrhythmia	8.02	9.16	9.24	9.13	8.39	7.41
Diabetes, no complications	7.22	8.70	7.73	8.01	7.47	6.81
Chronic lung disease	5.38	6.13	6.39	5.67	5.35	5.12
Hypothyroidism	4.32	4.21	4.22	4.24	4.24	4.39
Congestive heart disease	3.37	4.27	4.22	3.96	3.71	2.95
Real failure/insufficiency	3.13	3.92	3.88	3.46	3.46	2.80
Peripheral occlusive vascular disease	2.30	2.94	2.45	2.67	2.37	2.13
Disorders of the water and electrolyte balance as well as of the acid–base balance	1.97	2.25	2.19	2.16	1.94	1.88
Other neurologic diseases	1.92	2.35	2.00	2.17	2.04	1.78
Heart valve disease	1.82	2.18	2.08	2.20	1.85	1.67
Coagulopathy	1.68	1.92	1.96	1.88	1.75	1.56
Depression	1.66	2.20	1.86	1.83	1.70	1.54
Hypertension, with complications	1.55	1.92	1.74	1.79	1.74	1.38

^a^Comorbidities with a total incidence of less than 1% are not presented (diabetes with complications, liver disease, alcohol abuse, paralysis, rheumatoid disease, psychosis, pulmonary heart disease and diseases of the pulmonary circulation, weight loss, deficiency anemia, drug abuse, and non-bleeding peptic ulcer)

### Endpoints

Table 4 illustrates the endpoint frequencies. A surgical complication within 90 days occurred in 4.2% of patients and recurrence operation within 91–365 days was performed in 1.0% of cases. Chronic pain requiring treatment within 365 days was observed in 2.9% of patients. The mortality rate within 30 days of admission was 0.3%. All four endpoints exhibited a significant trend and declined in line with a rising caseload volume. The proportionately greatest decrease of 32.8% was observed in the recurrence operations. Their proportion declined from 1.4% in the lowest caseload to 0.9% in the highest caseload volume category.

**Table 4:** Unadjusted frequencies of the endpoint events studied, presented as a total figure and in accordance with volume categories (I: 1–50, II: 51–75, III: 76–100, IV: 101–125, V: ≥ 126 inguinal hernia repairs per year)

	Total (%)	I (%)	II (%)	III (%)	IV (%)	V (%)	P
Surgical complications (90 days)	4.22	4.49	4.61	4.55	4.10	4.11	0.001
Recurrence (91–365 days)	0.95	1.38	1.11	1.00	0.84	0.93	0.003
Pain management (365 days)	2.87	3.31	3.03	2.84	2.94	2.80	0.043
Mortality (30 days)	0.28	0.31	0.35	0.37	0.27	0.25	0.010

### Influence of the caseload volume

The results of logistic regression analysis are presented in Table 5. The hospital volume had a significant influence on the risk-adjusted recurrence rate. The risk of recurrence operation in the two lowest caseload categories (1–50 cases and 51–75 cases per year) compared with the highest caseload category was increased by 53% and 24%, respectively (OR: 1.53 and 1.24). Had these cases been treated in the same way as the highest caseload category 22 (36.1%) and 27 (20.9%), respectively, of recurrence operations could possibly have been prevented in these categories (NNT: 212 and 451, respectively). The decreasing odds ratios in line with increasing caseload point to a linear volume-outcome relationship. Other factors associated with an increasing risk were, e.g., the initial recurrence status, bilateral operation and simultaneous repair of a femoral hernia, drug abuse, and disorders of the water and electrolyte balance as well as of the acid–base balance. The use of a mesh procedure as well as patient age ≥ 76 years reduced the risk of recurrence procedure.

**Table 5:** Logistic regression analysis for assessment of the influencing factors (odds ratio) on the endpoints investigated

Influencing factors	Surgical complications (90 days)	Recurrence procedure (91–365 days)	Pain management (365 days)	Mortality (30 days)
Volume categories (caseload annual)				
I (1–50)	0.94 (0.79–1.13)	1.53 (1.17–1.98)	1.09 (0.93–1.28)	1.00 (0.55–1.82)
II (51–75)	1.01 (0.90–1.14)	1.24 (1.01–1.55)	1.03 (0.91–1.16)	1.06 (0.71–1.58)
III (76–100)	1.01 (0.91–1.12)	1.10 (0.91–1.34)	0.97 (0.87–1.07)	1.11 (0.82–1.50)
IV (101–125)	0.94 (0.85–1.05)	0.93 (0.77–1.12)	1.02 (0.93–1.12)	0.83 (0.59–1.17)
V (≥ 126)	1 (reference)	1 (reference)	1 (reference)	1 (reference)
Age in years				
18–44	1 (reference)	1 (reference)	1 (reference)	1 (reference)
45–55	1.20 (1.07–1.35)	–	1.93 (1.69–2.21)	–
56–64	1.31 (1.16–1.49)	–	2.05 (1.79–2.35)	–
65–75	1.51 (1.35–1.69)	–	1.96 (1.71–2.25)	4.98 (2.95–8.41)
76–102	1.81 (1.60–2.04)	0.78 (0.66–0.92)	2.46 (2.14–2.83)	13.7 (8.47–22.16)
Risk factors and surgical technique				
Gender (female)	–	–	1.54 (1.40–1.68)	–
Recurrence present on admission	1.32 (1.22–1.44)	1.36 (1.17–1.59)	1.20 (1.10–1.32)	–
Incarceration	1.71 (1.58–1.85)	–	–	2.40 (1.73–3.33)
Gangrene	2.17 (1.62–2.89)	–	–	2.77 (1.46–5.25)
Emergency	–	–	–	1.86 (1.32–2.63)
Bilateral operation	1.23 (1.12–1.35)	1.58 (1.38–1.81)	1.12 (1.02–1.24)	–
Intestinal procedure	3.77 (2.78–5.12)	–	–	1.97 (1.17–3.30)
Umbilical hernia repair	–	–	–	2.03 (1.16–3.55)
Femoral hernia repair	1.51 (1.05–2.17)	2.12 (1.12 – 4.00)	–	–
Incisional hernia repair	1.78 (1.22–2.58)	–	–	–
Preoperative therapy with systemic glucocorticoids	1.29 (1.09–1.52)	–	2.04 (1.70–2.46)	2.15 (1.35–3.41)
Preoperative antithrombotic therapy	1.11 (1.01–1.21)	–	1.16 (1.06–1.28)	–
Day care surgical repair	–	–	–	0.09 (0.01–0.62)
Open mesh procedure	0.63 (0.51–0.78)	0.42 (0.34–0.52)	–	0.52 (0.39–0.70)
Minimally invasive mesh procedure	0.48 (0.38–0.60)	0.51 (0.41–0.62)	0.91 (0.84–0.98)	0.22 (0.15–0.33)
BMI and Elixhauser comorbidities				
Grade II obesity	1.63 (1.30–2.06)	–	1.70 (1.32–2.20)	–
Grade III obesity	3.25 (2.41–4.40)	–	2.49 (1.72–3.60)	–
Grade obesity unspecified	–	–	1.65 (1.06–2.55)	–
Alcohol abuse	–	–	2.05 (1.55–2.73)	–
Cardiac arrhythmia	1.36 (1.23–1.50)	–	–	1.63 (1.25–2.12)
Congestive heart disease	1.17 (1.03–1.32)	–	1.34 (1.16–1.54)	2.41 (1.79–3.24)
Coagulopathy	2.51 (2.20–2.86)	–	–	2.27 (1.62–3.17)
Chronic lung disease	–	–	1.51 (1.35–1.70)	–
Deficiency anemia	1.70 (1.17–2.47)	–	–	–
Depression	1.43 (1.20–1.71)	–	1.39 (1.13–1.71)	–
Diabetes, with complications	–	–	1.39 (1.04–1.86)	–
Diabetes, no complications	1.13 (1.03–1.24)	–	1.21 (1.09–1.35)	–
Drug abuse	–	3.43 (1.61–7.28)	2.40 (1.40–4.13)	–
Disorders of the water and electrolyte balance as well as of the acid–base balance	3.04 (2.65–3.48)	1.65 (1.17–2.32)	1.26 (1.05–1.51)	3.73 (2.82–4.95)
Hypertension, with complications	0.76 (0.61–0.94)	–	–	0.38 (0.23–0.63)
Hypertension, no complications	–	–	1.18 (1.09–1.28)	0.59 (0.47–0.75)
Liver disease	2.33 (1.89–2.86)	–	–	4.86 (2.99–7.91)
Other neurologic diseases	–	–	–	1.62 (1.05–2.52)
Paralysis	–	–	1.49 (1.09–2.03)	–
Pulmonary heart disease and diseases of the pulmonary circulation	1.61 (1.18–2.19)	–	–	1.81 (1.04–3.14)
Peripheral occlusive vascular disease	–	–	1.41 (1.19–1.67)	–
Real failure/insufficiency	1.26 (1.12–1.43)	–	–	1.52 (1.14–2.04)
Rheumatoid disease	–	–	2.02 (1.52–2.68)	–
Heart valve disease	1.26 (1.07–1.49)	–	–	–
Weight loss	1.94 (1.34–2.82)	–	–	2.40 (1.29–4.46)

Risk factors denoted by “–“were not included in risk adjustment because of a lack of significance

For the endpoints surgical complications within 90 days, chronic pain requiring treatment within 365 days and 30-day mortality, the hospital volume was not found to have any significant influence. Factors that greatly increased the risk of surgical complications were the presence of gangrene, an intestinal procedure, grade III obesity or disorders of the water and electrolyte balance as well as of the acid–base balance. For chronic pain requiring treatment the factors patient age ≥ 76 years, grade III obesity and drug abuse exhibited a strong risk-increasing impact. As regards the 30-day mortality, patient age (≥ 76 and 65–75 years) had by far the greatest influence. Other risk-increasing factors were the presence of gangrene, liver diseases, and disorders of the water and electrolyte balance as well as of the acid–base balance.

We calculated VIFs for all covariates of the presented models. Table 6 shows the mean and maximum VIF for each model. With the exception of one covariate used in the model for surgical complications all VIFs were below 3. These results indicate that multicollinearity is not an issue. Especially not in the case of recurrence procedures. In addition not all covariates listed in Table 3 were present in each model. The footnote of Table 5 indicates that the specific covariate was removed from model due to lack of significance. For example, in the model of recurrence procedures only 12 covariates were included.

**Table 6:** Mean and maximal variance inflation factor of covariates used within the presented multiple logistic regression models

Logistic model	Mean VIF	Maximal VIF of a single predictor
Surgical complications	1.37	3.33
Recurrence procedure	1.23	1.75
Pain management	1.23	2.06
Mortality	1.33	2.16

## Discussion

This study investigated the relationship between hospital volume and outcome on the basis of 133,449 inguinal hernia repairs from 1060 hospitals. The analysis demonstrated that caseload volume had a significant influence on the endpoint recurrence operation within 91–365 days. The risk of recurrence operation was significantly increased in hospitals with less than 76 inguinal hernia repairs per year. The hospital volume had no impact on the other endpoints studied.

The volume-outcome correlation identified for recurrence operations concords with the findings of the international studies cited above. These investigated both the influence of the surgeon volume [8, 10–13] and the influence of the hospital volume [9, 14]. Using five volume categories it was possible to confirm the existing volume impact at a cutoff point of 50 procedures per year [9] as well as absence of such volume effect at a cutoff point of 140 procedures per year [14]. In the present study already for 76 procedures per year no influence of the hospital volume could be detected. For the surgeon volume Köckerling et al. and Aquina et al. each reported a cutoff point of 25 cases per year based on laparoscopic [12] and open surgical procedures [14]. While these cutoff points are essentially lower, they concord with the findings reported here since in general a hospital’s caseload is distributed across several surgeons and surgical techniques. However, hospital volume should not be treated as equivalent to surgeon volume. It rather addresses the experience of the entire treatment chain within a hospital. This includes not only the experience of the surgeon but also the experience of, e.g., surgical assistances, nurses, post operational treatment and material management.

In addition to demonstrating the complication risks in relation to the caseload volume, the present study investigated the implications of reassignment of patients from the categories with significantly increased complication risks to the highest caseload category. In the low caseload categories it would have been possible theoretically to prevent one-out-of-every three and one-out-of-every five recurrence operations, respectively. However, in total that relates to only 59 recurrence operations since the caseloads in these categories, accounting for a total proportion of 12.5%, were markedly smaller.

Other endpoints were investigated individually in the study by Köckerling et al. [12]. As in the present study, the authors did not identify any volume-outcome correlations for chronic pain requiring treatment or for peri- and postoperative complications, comparable with the surgical complications’ endpoint employed in this present study.

The different distribution of surgical techniques observed in relation to the caseload volume has also been reported in the literature [8, 14]. Minimally invasive surgical techniques, in particular, have greater complexity and may therefore be used more often in the high caseload hospitals. Additionally we observed that patients in low volume hospitals tend to have more comorbidities and thus might be more difficult to treat. However, regarding the presented volume effects all patient specific characteristic were taken into account by the multiple regression analysis if they had a significant influence. Our results indicate that especially those patients should be treated in experienced hospitals to avoid complications. To determine why these patients were treated more often by low volume hospitals more research is needed.

## Limitations

The study has a number of limitations. First, it is based on secondary analysis of routine data. Under- or overdocumentation of individual events cannot be ruled out. Furthermore, only events reflected in the catalog systems could be evaluated. The OPS catalog made no provision for differentiation between the various surgical techniques. Nor can the size and location of the inguinal hernia be documented on the basis of the catalog systems. There are also limitations with regard to external validity of the patient characteristics and endpoint frequencies since the patient collective studied was composed exclusively of AOK-insured persons. Although the collective of AOK-insured persons accounts for more than one-third of hospital cases in Germany, there are certain differences versus the population of persons insured by other statutory sickness funds in terms of the age structure and comorbidity profile [24]. Besides, this study includes only in- and outpatient inguinal hernia repairs performed in hospitals with no account taken of outpatient repairs performed by contractual statutory health insurance surgeons outside hospitals.

## Conclusion

The present study of current data demonstrates a clear correlation between hospital volume and the recurrence operation rate following inguinal hernia repairs. Hospitals with less than 51 and 76 inguinal hernia repairs per year have a significantly higher risk of recurrence operations. One-out-of-every three and one-out-of-every five recurrence operations for patients in these hospitals could possibly have been avoided had these patients been operated on in high volume hospitals. Therefore, from a patient’s perspective the number of inguinal hernia repairs performed per year can serve as a guide to choosing a hospital. For the additional endpoints investigated, i.e., chronic pain requiring treatment, surgical complications and 30-day mortality, no correlation was identified between the hospital volume and complication rate.
